# Predictive factors of first dosage intravenous immunoglobulin-related adverse effects in children

**DOI:** 10.1371/journal.pone.0227796

**Published:** 2020-01-13

**Authors:** Jun Kubota, Shin-ichiro Hamano, Atsuro Daida, Erika Hiwatari, Satoru Ikemoto, Yuko Hirata, Ryuki Matsuura, Daishi Hirano

**Affiliations:** 1 Division of Neurology, Saitama Children's Medical Center, Saitama, Japan; 2 Department of Pediatrics, The Jikei University School of Medicine, Tokyo, Japan; 3 Department for Child Health and Human Development, Saitama Children's Medical Center, Saitama, Japan; Heinrich-Heine-Universitat Dusseldorf, GERMANY

## Abstract

**Background:**

Intravenous immunoglobulin (IVIG) therapy is used in the treatment of various diseases, and IVIG-related adverse effects (IVIG-AEs) vary from mild to severe. However, the mechanisms underlying IVIG-AEs and the potential predictive factors are not clear. This study investigated whether certain IVIG-AEs can be predicted before IVIG administration.

**Study design and methods:**

This retrospective cohort study at the Division of Neurology, Saitama Children’s Medical Center included patients enrolled from 2008 to 2018 who were < 18 years old and received IVIG for the first time. IVIG-AEs were classified according to the Common Terminology Criteria for Adverse Events version 5.0.

**Results:**

A total of 104 patients fulfilled the inclusion criteria. The rate of IVIG-AEs was 37.5% (39/104). The most frequent IVIG-AEs were fever (41.0% [16/39]) and headache (38.5% [15/39]). AEs were below grade 2 in all except one patient and there were no grade 4 AEs. High serum total protein (TP) level was significantly related to the occurrence of IVIG-AEs (odds ratio, 14.8; 95% confidence interval, 2.4–90.5; *P* < 0.01). The optimal cutoff TP level was 6.7 g/dL. Although low WBC count and immunoglobulin G level may be predictive risk factors of IVIG-AEs, it was not confirmed in this study.

**Conclusion:**

IVIG-AEs occurred in 37.5% of cases, and most were mild. TP was the best predictive risk factor of IVIG-AEs before IVIG administration. These results may aid in elucidating the mechanism underlying IVIG-AEs.

## Introduction

Intravenous immunoglobulin (IVIG) therapy is widely used in the treatment of primary and secondary immunodeficiency diseases, Kawasaki disease, idiopathic thrombocytopenic purpura, and neurological diseases, such as Guillain–Barré syndrome, chronic inflammatory demyelinating polyneuropathy, myasthenia gravis, polymyositis, multiple sclerosis, autoimmune encephalitis, and epilepsy [1,2]. Regardless of age, disease, and severity of adverse effects (AEs), the incidence of IVIG-related AEs (IVIG-AEs) has been reported to be 0.6% – 87.5% [1,3–13]. The incidences of IVIG-AEs were reported to be lower in children than in adults; however, they still range from less than 1% to 40% [3,12]. Three major issues arising from prior studies include the large heterogeneity of AE definitions, the relatively small sample sizes, and the large variation in underlying diseases. It is possible that the lower incidence of IVIG-AEs in children results from the fact that children, especially infants, cannot complain of subjective IVIG-AEs such as headache, nausea, and abdominal pain.

IVIG-AEs are divided into immediate AEs that occur during or within 30 minutes after starting IVIG administration and delayed AEs that occur from 8 hours to 10 days after commencement of IVIG administration [1,3,5,13,14]. In a previous prospective study of IVIG-AEs in pediatric patients, the incidence rates of immediate and delayed AEs were reported to be 10.3% and 41.4%, respectively [3]. Although delayed AEs were reported to be more common IVIG-AEs in children, this study evaluated only 58 cases, with a small total number of infusions (345 infusions) [3]. Immediate AEs in adult and pediatric patients show mild flu-like symptoms, such as headache, flushing of the face, malaise, tightness in the chest, fever, chills, myalgia, fatigue, dyspnea, back pain, nausea, and tachycardia [1,3,5,13,14]. In contrast, the symptoms of delayed AEs include severe symptoms, such as acute renal failure, thromboembolic events, neurological toxicity (i.e., aseptic meningitis), hematological toxicity, dermatological toxicity, pseudohyponatremia, arthritis, and pulmonary complications [1,3,5,13,14]. Several previous studies suggested that migraine may be a risk factor for aseptic meningitis associated with IVIG [4,15,16]. Other studies suggested that IVIG infusion rate, primary infusion of IVIG, history of IVIG-AEs, hydration before and after IVIG infusion, immunoglobulin preparation, underlying diseases (immunoglobulin A deficiency, hypertension, thrombopoiesis, etc.), and age may be risk factors of IVIG-AEs [1,6,7,13,14,17,18]. However, clinical data regarding predictive risk factors for IVIG-AEs are limited. The mechanisms underlying IVIG-AEs, including both immediate and delayed AEs, have yet to be elucidated.

It can be difficult to distinguish between exacerbation of underlying disease and IVIG-AEs, especially in children, because of the similarity of some symptoms, such as headache, vomiting, nausea, and fever. Depending on the symptoms of IVIG-AEs, slowing the IVIG infusion rate or discontinuing IVIG may prolong the treatment period and thus lead to an extended period of hospitalization. The ability to predict patients at high risk of IVIG-AEs prior to administration of IVIG would make it possible to prevent or reduce the incidence of these effects. In addition, identifying predictive factors of IVIG-AEs may provide insight into the underlying mechanisms responsible for these events.

This study was performed to determine whether it is possible to predict IVIG-AEs in pediatric patients including both immediate and delayed AEs, based on clinical data collected before administration of the first dose of IVIG.

## Materials and methods

### Study design and patients

This was a retrospective cohort study performed at Saitama Children’s Medical Center, Saitama, Japan.

Patients < 18 years old treated with at least one infusion of IVIG during admission to the Division of Neurology, Saitama Children’s Medical Center between February 1, 2008, and March 31, 2018, were considered for inclusion in the study. The exclusion criteria were previous IVIG therapy, application of IVIG therapy for infection, no laboratory examination 14 days before commencement of IVIG, prednisolone treatment, and history of methylprednisolone pulse or adrenocorticotropic hormone treatment before IVIG. As previous studies suggested that corticosteroid may be related to the reduction of IVIG-AEs [1,9,13,14], patients that had received corticosteroids were also excluded from the present study.

Sex, age, underlying disease, laboratory data (white blood cell (WBC) count, total protein (TP), blood urea nitrogen (BUN), sodium (Na), glucose, immunoglobulin A (IgA), immunoglobulin G (IgG), and immunoglobulin M (IgM)), calculated osmotic pressure (Na × 2 + glucose/18 + BUN/2.8) [19], immunoglobulin preparation, dosage of IVIG (g/kg), duration of IVIG therapy (days), change in infusion rate (0.6 mL/kg/h during the first 30 minutes to 1 hour, and 1.8 mL/kg/h subsequently), and hydration around and/or during IVIG administration were evaluated. When IVIG was discontinued owing to AEs, IVIG dosage was defined as the total dose after administration if IVIG-AEs occurred outside the period of IVIG administration, or including the dose planned for the day if IVIG-AEs occurred during the period of IVIG administration. The duration of IVIG therapy included the day of AE onset.

Underlying diseases were divided into five categories: epilepsy, central nervous system disease (encephalitis/encephalopathy, cerebellitis, acute disseminated encephalomyelitis, and clinically isolated syndrome), peripheral nervous system disease (including chronic inflammatory demyelinating polyneuropathy, Guillain-Barré syndrome, Miller–Fisher syndrome, and myelitis), myasthenia gravis, and hypogammaglobulinemia.

Three immunoglobulin preparations were used in this study: Venoglobulin IH 5%, Kenketsu Glovenin-I, and Kenketsu Venilon-I (Table 1).

**Table 1:** Immunoglobulin preparations.

	Venoglobulin IH 5%	Kenketsu Glovenin-I	Kenketsu Venilon-I
Manufacturer	Japan Blood Products Organization	Nihon Pharmaceutical Co., Ltd.	Teijin Pharma Ltd.
Form	Liquid	Lyophilized. Cohn-Oncley	Lyophilized
Method of preparation (including viral inactivation)	Pasteurization (60°C, 10 h), low-pH incubation, nanofiltration	Polyethylene glycol, ion-exchange, nanofiltration	Sulfonation, nanofiltration (virus removal membrane 19 nm)
Shelf-life/storage requirements	24 months/not more than 10°C, do not freeze	24 months	24 months/storage at room temperature
Preparation time	Immediately	Unknown, must be dissolved	Dissolution time 3 minutes (2.8 ± 0.5 minutes): 2.5 g product
Sugar content	D-Sorbitol: 47.4 mg/mL	0	0.9% (9 mg/mL)
Sodium content	2.6 mEq/L	391.3 mEq/L (9 mg/mL)	171 mEq/L
Stabilizer	D-Sorbitol	Glycine, D-mannitol, NaCl	Glycine, D-Mannitol
Potential for TSE/prion removal	Yes	Yes	> log10^4.3^
pH	3.9–4.4	6.4–7.2	6.4–7.2
Osmolality	Approx. 1 (ratio with physiological saline)	400–523 mOsm/kg	Approx. 2 (ratio with physiological saline)
Albumin	Not detected	Not available	0.0025% (0.25 mg/mL)
IgA content	Below detection level	27 μg/mL (3 lot mean)	5.0 ± 1.4 mg/dL
IgG	99.80%	> 99%	90–110%
Latex content in packaging	Not used	Not used	Not used

IgA, immunoglobulin A; IgG, immunoglobulin G; TSE, transmissible spongiform encephalopathy.

### Outcome measures

The primary endpoints were the occurrence of AEs from the first day of IVIG administration to 7 days after the end of IVIG administration.

### Definition of adverse effects

IVIG-AEs were defined as symptoms appearing from the first day of IVIG administration to 7 days after the end of IVIG administration. Immediate AEs were defined as symptoms occurring during infusion, and delayed AEs were defined as symptoms occurring after the infusion has ceased [3]. The symptoms of IVIG-AEs were classified according to the Common Terminology Criteria for Adverse Events (CTCAE) version 5.0. [20]. Subjective IVIG-AEs such as headache and abdominal pain were evaluated as much as possible by interviewing guardians if patients could not provide any information on subjective IVIG-AEs. Besides, the Face, Legs, Activity, Cry, and Consolability (FLACC) [21–23] or revised FLACC (r-FLACC) Scale [24,25] was used for those patients. The guardians rated patients’ pain at its highest stage in each category on a scale of 0 to 2, yielding an overall pain score of 0‒10 [21–25]. Then, FLACC scores 1–3, 4–6, and 7–10 were classified based on the CTCAE of pain categories, such as headache and abdominal pain, into grades 1, 2 and 3, respectively.

The first day of IVIG administration was defined as day 1. We evaluated AEs every 24 hours until 7 days after the end of IVIG administration. All AEs that occurred during this period were evaluated. When multiple AEs occurred, the first day of AE occurrence was considered the IVIG-AE onset day.

### Statistical analysis

Continuous variables are expressed as the median and interquartile range, whereas categorical variables are expressed as frequencies. We used the non-parametric Mann–Whitney *U* test for comparisons of continuous variables between the two groups, AE and non-AE, and the chi-square or Fisher’s exact test, as appropriate, for analysis of categorical data. Multivariate regression analysis was performed using logistic regression analysis for sex, age, and data that showed significant differences in univariate analysis. Forward stepwise regression analysis was performed for sex, age, and data that showed significant differences in univariate analysis. The stepwise procedure was set using a threshold of 0.05 for inclusion. The cutoff point was determined according to the Youden Index [26] based on the receiver operating characteristic (ROC) curve for data that showed significant differences in multivariate regression analysis.

Statistical analyses were performed using Stata version 15.1 (Stata Corp., College Station, TX). In all analyses, *P* < 0.05 was taken to indicate statistical significance.

This study was conducted in accordance with the ethical principles of the Declaration of Helsinki and with the ethical guidelines for epidemiological studies issued by the Ministry of Health, Labour and Welfare, Japan. This study was approved by the Saitama Children’s Medical Center Institutional Review Board (2018-02-008). Informed consent was not deemed necessary because the data were obtained retrospectively from the patient charts.

## Results

### Baseline patient characteristics and infusion features

**Fig 1:**
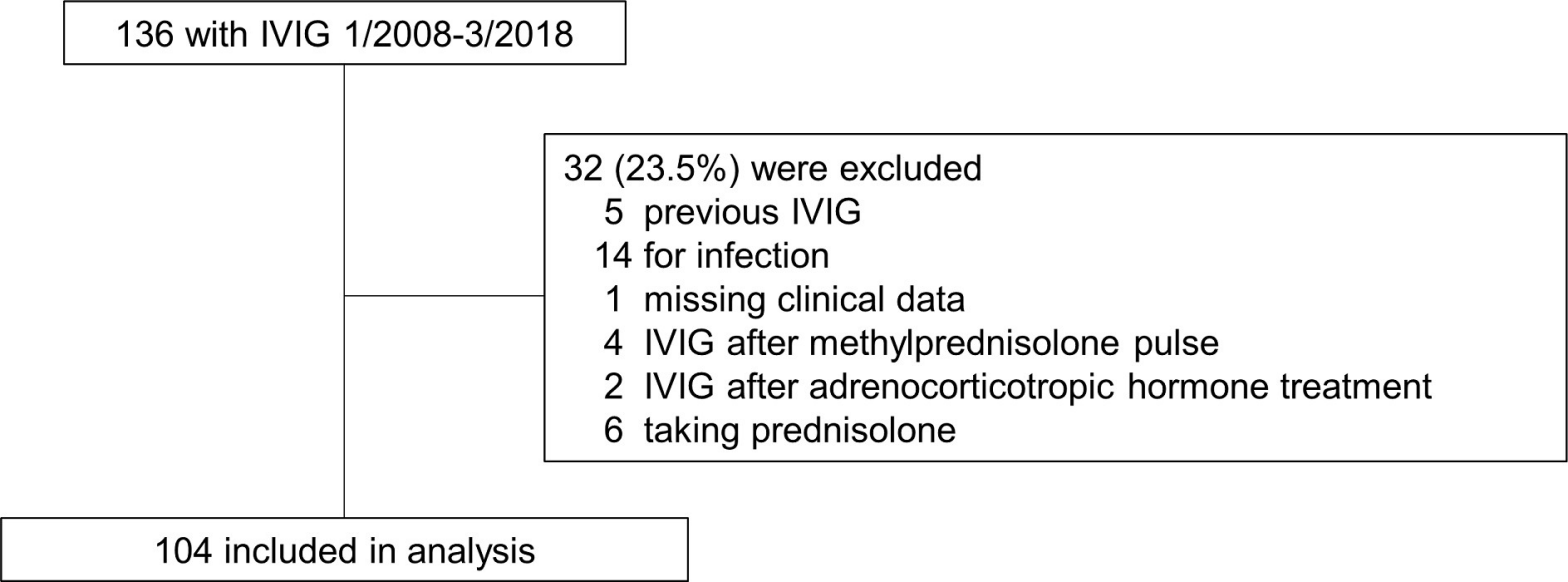
Patients included and excluded from the study. IVIG, intravenous immunoglobulin.

A total of 136 children underwent IVIG during the study period at our institute, and 104 (76.5%) of these children fulfilled the inclusion criteria (Fig 1). The baseline characteristics of the patients are shown in Table 2. Dataset is available as Supporting information (S1 Database). The median age of the study population was 8.5 months (interquartile range [IQR] 6 – 54.5 months), and 52.9% were male. The most common underlying disease was epilepsy (66.3%). The median total dosage of IVIG was 1.08 g/kg (IQR 0.86 – 1.25 g/kg) and the median duration of IVIG therapy was 3 days (IQR 3 – 5 days). The most frequently selected immunoglobulin preparation was Venoglobulin IH 5% (43.3%). The median first and subsequent infusion rates were 0.56 mL/kg/h (IQR 0.51–0.60 mL/kg/h) and 1.70 mL/kg/h (IQR 1.52–1.79 mL/kg/h), respectively.

**Table 2:** Baseline characteristics of patients.

	Total (n = 104)	Adverse effects (n = 39)	Non-adverse effects (n = 65)
Sex (male : female)	55:49	20:19	35:30
Age [month], median (IQR)	8.5 (6–54.5)	51 (8–91)*	7 (6–13)*
Disease classification			
Epilepsy	69	19*	50*
Central nervous system disease†	12	5	7
Peripheral nervous system disease‡	13	9**	4**
Myasthenia gravis	8	6	2
Hypogammaglobulinemia	2	0	2
Total IVIG [g/kg], median (IQR)	1.08 (0.86–1.25)	1.06 (0.75–1.60)	1.08 (0.86–1.21)
Duration of IVIG therapy [days], median (IQR)	3 (3–5)	4 (3–5)*	3 (3–5)*
Immunoglobulin preparations			
Venoglobulin IH 5%	45	21	24
Kenketsu Glovenin-I	40	12	28
Kenketsu Venilon-I	19	6	13
Change of infusion rate (n)	99	38	61
First infusion rate [mL/kg/h], median (IQR)	0.56 (0.51–0.60)	0.55 (0.51–0.61)	0.57 (0.51–0.60)
Subsequent infusion rate [mL/kg/h], median (IQR)	1.70 (1.52–1.79)	1.71 (1.52–1.83)	1.69 (1.53–1.79)
Hydration around and/or during IVIG administration (n)	19	7	12
Laboratory data check days [day], median (IQR)	1 (0–5)	1 (0–5)	1 (0–6)
WBC [/μL], median (IQR)	8200 (6950–11150)	7400 (6200–8800)*	10000 (7500–12500)*
TP [g/dL], median (IQR)	6.4 (5.8–6.8)	6.8 (6.1–7.0)*	6.1 (5.7–6.6)*
BUN [mg/dL], median (IQR)	9 (6–12)	10 (8–13)*	8 (6–11)*
Na [mmol/L], median (IQR)	138 (137–140)	139 (138–141)*	138 (137–139)*
Glucose [mg/dL], median (IQR)	94 (87–103)	94.5 (86–103)	93 (87–103)
IgG [mg/dL], median (IQR)	591.5 (379.5–840.5)	754 (505–912)*	478.5 (361.0–703.0)*
IgA [mg/dL], median (IQR)	32 (16–74)	62 (25–101)*	30 (15–52)*
IgM [mg/dL], median (IQR)	62 (45–93)	71 (45–117)	60.5 (47.0–86.0)
Calculated osmotic pressure [mOsm/kg H2O], median (IQR)	285.1 (282.6–288.5)	287.8 (283.9–290.2)*	284.7 (282.0–287.8)*

* P < 0.01

** P < 0.05

†Central nervous system disease total number (number of adverse effects) = encephalitis/encephalopathy 8 (3), cerebellitis 2 (0), acute disseminated encephalomyelitis 1 (1) and clinically isolated syndrome 1 (1)

‡Peripheral nervous system disease total number (number of adverse effects) = chronic inflammatory demyelinating polyneuropathy 7 (6), Guillain–Barré syndrome 3 (2), Miller–Fisher syndrome 1 (0), myelitis 1 (0), and others 1 (0)

BUN, blood urea nitrogen; IgA, immunoglobulin A; IgG, immunoglobulin G; IgM, immunoglobulin M; IQR, Interquartile range; IVIG, intravenous immunoglobulin; Na, sodium; TP, total protein; WBC, white blood cells.

The median values of laboratory data were as follows: WBC count, 8200/μL; TP, 6.4 g/dL; IgG, 591.5 mg/dL; IgA, 32 mg/dL; and calculated osmolality, 285.1 mOsm/kg H_2_O.

### Adverse effects

A total of 65 IVIG-AEs occurred in 39 patients (37.5%) during the study period. Table 3 shows the types of immediate and delayed AEs. The incidence of delayed AEs (79.5%, 31/39) was higher than that of immediate AEs (30.8%, 12/39). The most common IVIG-AE was fever. Only one of the patients had a grade 3 AE (headache), and there were no cases of a grade 4 AE. The incidence rate of fever in the study population was 41.0% (16/39). The second most common AE was headache, which had a rate of 38.5% (15/39). Maculopapular rash, vomiting, and nausea occurred with incidence rates of 33.3% (13/39), 25.6% (10/39), and 15.4% (6/39), respectively. The least common AE was abdominal pain, which occurred at a rate of 12.8% (5/39). IVIG-AEs occurred a median of 3 days (IQR, 2 – 4 days) after IVIG administration.

**Table 3:** Immediate and delayed adverse effects.

	Overall	Immediate	Delayed	Grade 1	Grade 2	Grade 3
Fever	16	4	12	14	2	0
Headache	15	6	12	11	3	1
Maculopapular rash	13	2	11	10	3	0
Vomiting	10	1	9	0	10	0
Nausea	6	1	5	3	3	0
Abdominal pain	5	1	5	5	0	0
Overall	65	15	54	43	21	1

AEs, adverse effects.

### Factors associated with the development of adverse effects

#### Univariate/multivariate analyses

The results of the univariate analysis of characteristics between patients with and without AEs are shown in Table 2. There were no statistically significant differences in sex, IVIG dosage, IVIG preparations, changes in infusion rate, hydration around and/or during IVIG administration, glucose, or IgM between the groups. However, the group with AEs showed significant associations with older age, diseases such as epilepsy and peripheral nervous system diseases, longer duration of IVIG therapy, lower WBC count, and higher TP, BUN, Na, IgG, IgA, and calculated osmotic pressure.

After adjustment for possible confounding factors, post-administration AEs showed significant associations with TP (odds ratio [OR], 14.8112; 95% confidence interval [CI], 2.4244–90.4840; *P* < 0.01), WBC count (OR, 0.9995; 95% CI, 0.9993–0.9998; *P* < 0.01), and IgG (OR, 0.9943; 95% CI, 0.9902–0.9984; *P* < 0.01) (Table 4).

**Table 4:** Multivariate regression analysis for predictive risk factors of IVIG-AEs.

	Odds Ratio (95% Confidence Interval)	*P* value
Male	0.9598 (0.2914–3.1616)	0.95
Infant (age < 12 months)	2.0760 (0.2814–15.3135)	0.47
Epilepsy	2.6187 (0.4492–15.2653)	0.28
WBC	0.9995 (0.9993–0.9998)	< 0.01
TP	14.8112 (2.4244–90.4840)	< 0.01
IgG	0.9943 (0.9902–0.9984)	< 0.01
IgA	1.0146 (0.9926–1.0371)	0.20
Calculated osmotic pressure	1.0666 (0.9115–1.2481)	0.42

IgA, immunoglobulin A; IgG, immunoglobulin G; IVIG, intravenous immunoglobulin; IVIG-AEs, IVIG-related adverse effects; TP, total protein; WBC, white blood cells.

#### Forward stepwise regression analysis

AEs showed significant associations with TP (OR, 15.3185; 95% CI, 2.6720–87.8202; *P* < 0.01), WBC count (OR, 0.9995; 95% CI, 0.9993–0.9998; *P* < 0.01), and IgG (OR, 0.9963; 95% CI, 0.9929–0.9996; *P* < 0.01). These results are consistent with multivariate analysis.

#### Optimal cutoff point for dichotomization

**Fig 2:**
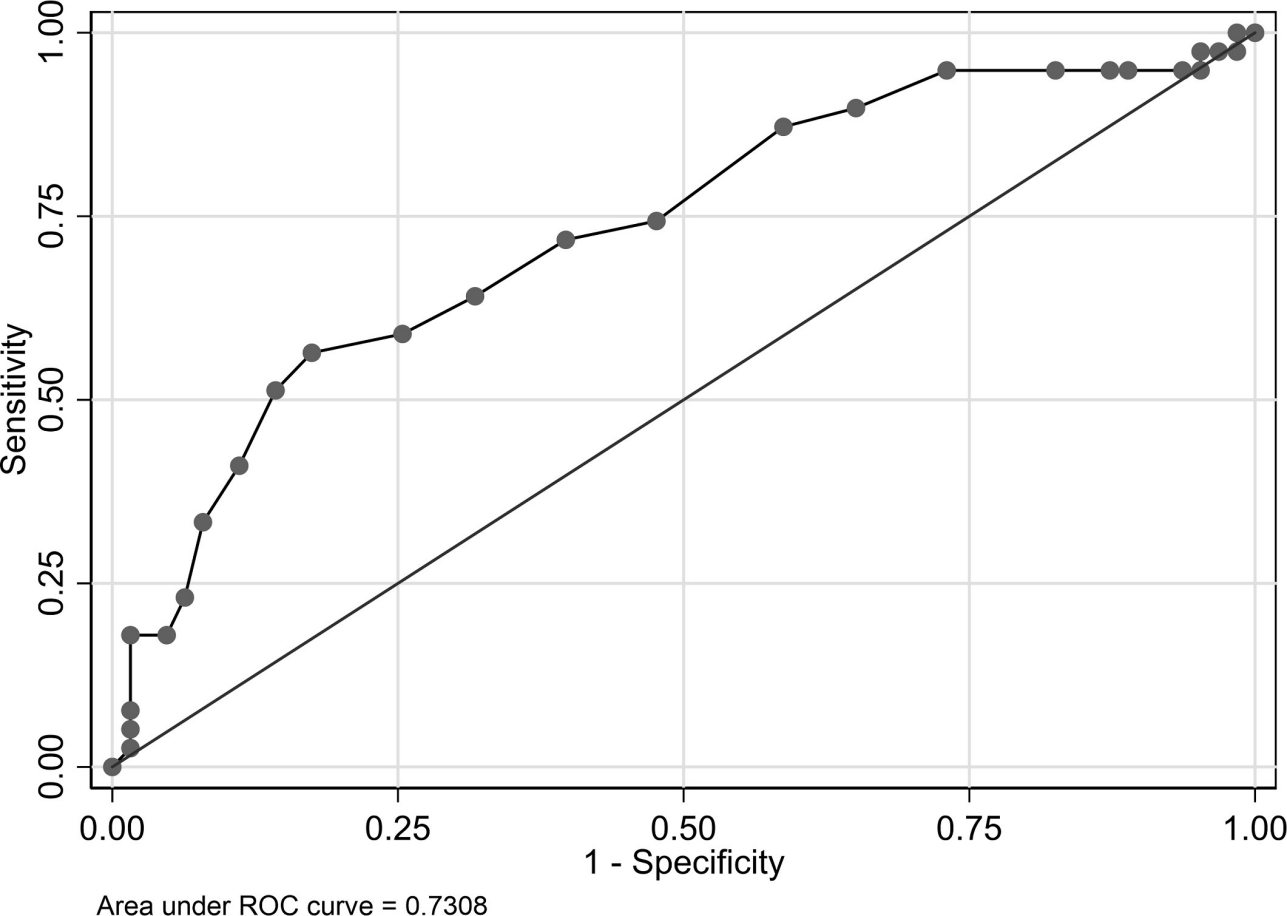
Receiver operating characteristic curve of total protein. The area under the ROC curve was 0.7308. ROC, receiver operating characteristic.

As shown in Fig 2, the area under the ROC curve for TP was 0.7308. ROC curve analysis indicated that the optimal cutoff point of TP was 6.7 g/dL.

## Discussion

This observational study was performed to investigate the incidence of IVIG-AEs in pediatric patients. In addition, predictive factors for the development of AEs were identified. To our knowledge, this was the largest study investigating IVIG-AEs to date and the first study investigating IVIG-AEs classified according to the CTCAE [20].

The incidence of IVIG-AEs in this study was 37.5%, and most were mild to moderate AEs (e.g., CTCAE grades 1 and 2) [20]. The incidences of IVIG-AEs in previous follow-up studies of IVIG infusion in pediatric patients ranged from 1% – 40% [3,12]. The major issues regarding these studies include the large degree of heterogeneity in AE definitions, relatively small sample sizes, and wide variation of underlying diseases. Limiting the underlying diseases to neurological diseases, IVIG-AEs were reported at rates of 13% – 21.4% in adults [9,11] and 23.5% in children [12]. The present study population included only patients receiving IVIG for the first time, whereas previous studies to evaluate IVIG-AEs included patients that had received IVIG several times. Several previous studies suggested that first-time IVIG therapy is one of the risk factors of IVIG-AEs [1,6,14]. It is reported that if IVIG-AEs occur with the first application of IVIG therapy, the occurrence of IVIG-AEs would significantly increase in subsequent IVIG therapies [27]. Therefore, further studies are required to determine the relations between the number of applications of IVIG therapy and IVIG-AEs.

In this study, IVIG-AEs occurred at a median of 3 days (IQR, 2 – 4 days) after start of IVIG administration, and most were delayed AEs. Singh-Grewal et al. reported that delayed AEs were more common than immediate AEs [3]. They suggested that the greater incidence of delayed AEs may have been owing to a lack of recognition of delayed AEs in the previous study, resulting in them being overlooked [3]. In addition, Markvardsen et al. reported that headaches are exacerbated on day 4 after IVIG [18]. Although the authors reported that most cases of delayed AEs have mild symptoms [3,18], other authors reported more severe symptoms associated with delayed as compared to immediate AEs [1,13,14]. This lack of recognition may result in delayed AEs being overlooked. Even with greater recognition of delayed AEs, most delayed AEs are likely to have mild symptoms as in the present study. However, it should be noted that there may be severe or even lethal events, such as thrombotic events, neurological disorders, renal impairment, hematological disorders, electrolyte disturbance, and transfusion-related infection, although their incidence rates may be low [1].

In this study, high serum TP level showed a significant relation to the occurrence of IVIG-AEs (OR, 14.8112; 95% CI, 2.4244–90.4840; *P* < 0.01), which was speculated to result from the hyperviscosity of the blood caused by the IVIG-induced increase in TP level. In fact, headache is known to be one of the symptoms of hyperviscosity syndrome [28,29]. Steinberger et al. prospectively administered IVIG at a dose of 2 g/kg for 2 – 5 days in cases of Guillain-Barré syndrome, multiple sclerosis, myasthenia gravis, immune cytopenia, and immunoglobulin deficiency, and examined changes in both serum TP level and viscosity before and after IVIG administration [19]. They reported that TP levels increased significantly at 6 and 24 hours after IVIG infusion, and the viscosity of the blood increased significantly at 24 hours after IVIG infusion (pre-IVIG, 6 hours post-IVIG, 24 hours post-IVIG: TP = 6.32, 7.10, and 8.15 g/dL, respectively; viscosity = 1.78, 1.89, and 1.98 to H_2_O, respectively) [19]. Bentley et al. also reported that plasma viscosity increases further after IVIG administration, especially as the number of IVIG administration days increases [30]. Therefore, higher TP levels before IVIG administration was suggested to be associated with an increase in viscosity of the blood after IVIG administration. The rates of IVIG-AEs were significantly greater in cases where the TP level was 6.7 or higher in the present study. Further prospective studies are required to determine whether this is the optimal TP cutoff point for predicting the occurrence of IVIG-AEs.

High WBC count and IgG level were significantly negatively correlated with the occurrence of IVIG-AEs in the present study (OR, 0.9995; 95% CI, 0.9993–0.9998; *P* < 0.01, OR, 0.9943; 95% CI, 0.9902–0.9984; *P* < 0.01, respectively). Although low WBC count and IgG level may be predictive risk factors of IVIG-AEs, it was not confirmed in this study. Future studies evaluating WBC count and IgG stratified according to age may explain their relations with IVIG-AEs and provide insights into the underlying mechanisms of IVIG-AEs.

This study had a number of limitations. First, the study population was small and limited to patients with neurological diseases. Therefore, the results may not be generalizable to other diseases. However, these results may help to distinguish between IVIG-AEs and exacerbation of underlying diseases at the onset of AEs because IVIG therapy is indicated for many neurological diseases. Further studies with a large sample size and addressing various diseases are needed. Second, IVIG-AEs may have been underestimated because of the occurrence of subjective IVIG-AE symptoms such as headache, nausea, and abdominal pain, which were evaluated by the physicians based on interviews with guardians. Therefore, IVIG-AEs may have been underestimated because infants and some patients with epilepsy or mental retardation would not have complained of subjective IVIG-AEs. IVIG-AEs were classified according to the CTCAE [20] in the present study to allow for the use of standardized definitions of IVIG-AEs which makes it possible to compare not only the results in future studies but also to compare AEs between different treatments. Third, only Japanese IVIG preparations were used in this study. However, there were no significant differences in the ingredients of these IVIG preparations compared to those available in other parts of the world [31,32] (Table 1). In addition, the composition of all three immunoglobulin preparations did not change during the study period. Therefore, the results of this study would be generalizable to IVIG preparations available in other countries. Fourth, the infusion rate of IVIG in all patients in this study was less than the maximum of 1.8 mL/kg/h, which was within the range described in the package inserts of Japanese IVIG preparations. Previous studies have shown that a maximum infusion rate for 5% IVIG preparations should be 4 mL/kg/h [7,14]. Although a slower infusion rate is associated with greater reduction in the incidence of IVIG-AEs [1,5,6,13,14], no significant association between infusion rate and occurrence of IVIG-AEs was observed in this study. Previous studies reported similar results [6,18]. Therefore, the relation between infusion rate and the occurrence of IVIG-AEs is still controversial. We could not stratify laboratory data by age because of the small sample size. The final limitation of the present study was that laboratory data were not evaluated after the end of IVIG administration and at the onset of IVIG-AEs. Such evaluations should be included in future studies.

At present, there is inadequate evidence for the efficacy of pretreatments for IVIG-AEs, and such treatments are controversial. Although pretreatments cannot prevent IVIG-AEs, some treatments, such as hydration, acetaminophen, nonsteroidal anti-inflammatory drugs, antihistamines, and corticosteroids, have been reported to reduce the incidence of IVIG-AEs [1,3,4,6,8,13–16,33]. It is difficult to distinguish between IVIG-AEs and exacerbation of underlying diseases, especially for relatively inexperienced physicians. A number of treatment options have been reported for when IVIG-AEs occur, such as decreasing the infusion rate or discontinuation of IVIG [6,13]; however, the occurrence of IVIG-AEs and reducing or stopping infusions would delay the treatment of underlying diseases. The identification of predictive factors for IVIG-AEs will allow comparative prospective studies in high-risk groups to develop effective pretreatments.

## Conclusions

The incidence of IVIG-AEs in first-time IVIG therapy was 37.5%, and most were mild-to-moderate delayed AEs. It is important to recognize the high incidence rate of delayed AEs. The occurrence of IVIG-AEs, especially headache, was strongly associated with TP levels ≥ 6.7 g/dL, owing to blood hyperviscosity. IVIG therapy is widely used for various diseases. Future studies should examine the validity of TP = 6.7 g/dL as a cutoff point for the prediction of IVIG-AEs. It will also be necessary to further identify the predictive factors and mechanisms underlying the occurrence of IVIG-AEs.

## Supporting information

S1 Database (XLSX)
